# Comparison of Lichtenstein Repair and Mesh Plug Repair Methods in The Treatment of Indirect Inguinal Hernia

**DOI:** 10.7759/cureus.2935

**Published:** 2018-07-06

**Authors:** Sebahattin Destek, Vahit Onur Gul

**Affiliations:** 1 General Surgery, Bezmialem Vakif University Faculty of Medicine, Istanbul, TUR; 2 General Surgery, Edremit State Hospital, Edremit/Balıkesir, TUR

**Keywords:** inguinal hernia, mesh-plug repair, lichtenstein repair

## Abstract

Objective: The lifetime risk to develop an inguinal hernia is 27%-43% for men and 3%-6% for women. Methods of hernia repair currently involve prosthetic mesh applications. The aim of this study is to compare the Lichtenstein repair and Mesh-plug repair methods in the surgical treatment of indirect inguinal hernias and to identify which of these two techniques is superior regarding its conferred advantages.

Materials and Methods: In this study, a total of 102 patients who were diagnosed with indirect inguinal hernia between the years 2014 and 2015 without a previous operation were analyzed. Patients undergoing Lichtenstein repair and Mesh-plug repair were compared, especially during operation time, hospital stay, postoperative pain and other aspects.

Results: The mean age of patients was 28.7 years (19-73). The mean duration of operations and hospitalizations was significantly shorter in patients who had undergone mesh-plug repair. Inguinal pain in the operation area on postoperative day one, two weeks and six months was significantly less in patients who had undergone mesh-plug repair. Patients were followed-up for two years.

Conclusion: We concluded that mesh-plug repair was superior to Lichtenstein repair regarding postoperative pain, quality of life of the patient, shorter duration of operation, and duration of hospital stay although the two methods were similar regarding both recurrence and complication rates. Considering this information, we suggest that mesh-plug repair can be used safely for the treatment of indirect inguinal hernias.

## Introduction

Lifetime occurrence of groin hernia is 27%-43% in men and 3%-6% in women [1]. A minority of patients are asymptomatic but even a watch-and-wait approach in this group results in surgery in approximately 70% within five years [1]. Worldwide, inguinal hernia repair is one of the most common surgeries, performed on more than 20 million people annually [1]. Although advanced techniques have been adopted in inguinal hernia repair parallel to those in developing medical technologies, currently no consensus has been reached on the best method among all existing methods [1].

In 1986, Lichtenstein used polypropylene mesh to strengthen the fascia transversalis and named the repair technique he developed as tension-free mesh repair [2]. Subsequently, mesh plug hernia repair was developed and was then adopted into common use. This technique has been widely performed at some centers in the USA with good results [3]. The mesh-plug repair (MPR) method is relatively simple compared with the Lichtenstein’s repair (LR) and involves placing a polypropylene mesh in the area of the defect [4]. The aim of a successful hernia repair is to achieve a minimal rate of recurrence while allowing the patient to return to normal activity in the shortest time with minimal discomfort.

In this study, we compared cases in which we performed either MPR or LR (Lichtenstein tension-free single flat of mesh) in patients with indirect inguinal hernia.

## Materials and methods

The study was designed as a prospectively randomized study. One hundred and two patients who were diagnosed to have indirect inguinal hernia between 2014 and 2015 years at Edremit Military Hospital and who had not been operated previously were included in the study. Patients younger than 18 years of age and patients with bilateral inguinal hernia and recurrent inguinal hernia were excluded from the study. The operations were explained to patients and consent was obtained. Both MPR and LR were performed in 52 and 50 patients, respectively, among the 102 patients. The same surgeon operated on all patients. These patients had a mean follow-up of two years.

Preoperatively, the two groups were evaluated regarding age, gender, body mass index (BMI), the presence of comorbidity, American Society of Anesthesiology (ASA) score, hernia type defined according to the Nyhus classification as determined by both superficial ultrasonography and site of the hernia. Superficial ultrasonography was performed at the radiology department using a superficial probe.

Patients were hospitalized in the morning on the day of the operation, and no preoperative medications were administered to any patient. The inguinal area of patients was shaved immediately preoperatively at the operating room. Six patients were given general anesthesia. Spinal anesthesia was performed in the other patients. No prophylactic antibiotics were administered.

Patients were compared with respect to the type of anesthesia, duration of operation, development of scrotal hematoma, and wound infection. The state of pain on postoperative day one, week one, and month one was assessed by a visual analog score (VAS) (0 = no pain, 10 = unbearable pain). Patients who underwent surgery, hospitalization, reduction in pain, duration of return to normal activity, and recurrence rates were evaluated. Patients were followed for a two-year risk of chronic pain and recurrence of hernia. Each patient underwent a physical examination to determine hernia recurrence. A superficial sonography was performed in the presence of a suspicious physical condition.

Surgical technique

The inguinal area of patients was shaved immediately preoperatively at the operating room following induction of anesthesia. Skin cleaning was performed using 10% povidone iodine; subsequently, the surgical field was covered with sterile drapes.

The operation started with an inguinal oblique incision. This incision was about 6 cm to 7 cm long and was made starting approximately 3 cm to 4 cm medial to the inguinal ligament and extending to the pubis. The incision was deepened, and the superficial layer of the subcutaneous fascia (Camper fascia) was entered. The fatty and aponeurotic tissue on the aponeurosis of the musculus obliquus externus was sharply dissected to expose the aponeurosis. After the aponeurosis had been cut, the lateral leaf was separated from the cord under it by blunt dissection up to the Poupart ligament. Subsequently, the medial leaf was separated by blunt dissection up to the conjoint tendon or transverse aponeurotic arch. Cremaster fibers were separated from the aponeurosis close to the pubis to mobilize the cord. External spermatic vessels and the genital branch of the genitofemoral nerve were preserved. The inguinal cord was suspended by a thin rubber drain. The inguinal sac was dissected from the pubis up to the internal ring, and the posterior wall was exposed. The ilioinguinal nerve was routinely preserved in this study.

**Figure 1:**
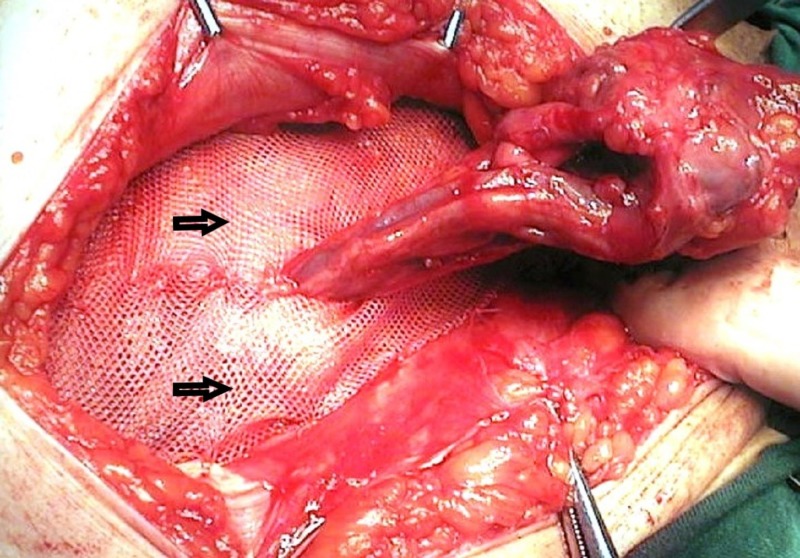
Lichtenstein repair, mesh placement.

For LR, the inguinal sac was tied using a high transfixion suture (2/0 polyglactin) and resected. Subsequently, a synthetic polypropylene mesh (L Prolene® mesh 10 x 15 cm; Ethicon Inc., Somerville, NJ, USA) was cut and prepared so that its edges would fit the posterior wall and its upper edge would cover the cord. Following placement of the mesh into its place, its lower edge was sutured to the pubic periosteum using 2/0 polypropylene suture. The synthetic polypropylene mesh was fixed to the lateral edge of the musculus rectus sheet and to the Poupart ligament on the other side. Mesh is usually fixed with intermittent suture. In patients, inguinal canal wall repair was not performed. (Figure 1) [1-2, 4].

**Figure 2:**
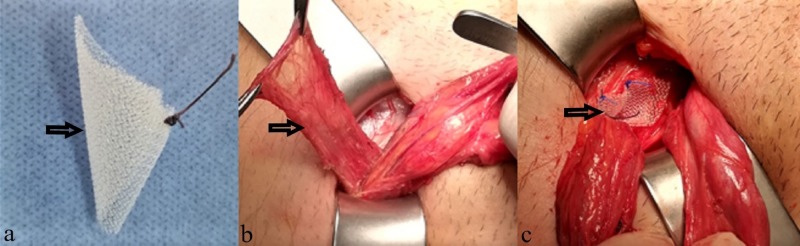
Mesh-plug repair. (a) Mesh plug (b) Hernia sac (c) Mesh-plug placement.

Unlike the LR group, the incision size was smaller (mean 3 cm-4 cm) in the MPR group and less dissection was performed to reveal the hernia sac. The cord was suspended. The indirect hernia sac was completely freed and reduced into the abdominal cavity. Subsequently, the same plain polypropylene mesh piece was longitudinally cut, folded like a cone or an umbrella, and thus formed a mesh plug. The mesh was inserted into the internal ring so that its narrow end remained inside. The mesh was placed on the transversalis fascia covering the plug. The latter was inserted into the inguinal ring. Interrupted 3/0 polypropylene sutures were used to fix the mesh in position. A mesh plug was applied in all patients using the Rutkow technique (Figure 2) [3]. 

Layers were closed in an anatomic plane in both groups following the repair. The aponeurosis of the external oblique muscle was sutured continuously using a 2/0 polypropylene suture, and the inguinal canal was reformed. Subcutaneous tissue and skin were closed primarily using 2/0 polyglactin and 3/0 polypropylene suture, respectively. The surgical field was cleaned with saline solution, and a dressing was applied at the end of the operation. The duration of the operation was recorded for all patients.

In the second postoperative hour, a single dose of diclofenac sodium (75 mg) was administered intramuscularly through the gluteal region as a standard analgesic medication during the postoperative period. Subsequently, patients received oral paracetamol 500 mg twice daily for seven days. On the first postoperative day, VAS was measured, and patients were questioned whether they required additional analgesics.

The time of discharge was recorded, and then an outpatient clinic follow-up was performed during the first postoperative week to evaluate VAS, possible complications, and duration of return to normal physical activity. Patients were invited to attend the outpatient clinic during the first and sixth postoperative months for follow-up visits.

Statistical analysis

The data obtained in this study were evaluated using SPSS package (SPSS Statistics for Windows, Version 20.0, IBM Corp, Armonk, NY) program. Frequency and percentage distributions of the data are presented. When comparing two groups, a Mann-Whitney U test was used for variables without a normal distribution as identified by a test for normal distribution. Following this, a Wilcoxon signed-rank test was used for non-normally distributed variables to compare pre- and post-measurement values. When evaluating differences between the groups, the level of significance used was 0.05, and a significant difference between the groups was reported when p < 0.05 while no significant difference was reported when p >0.05. Dependency between the variables was analyzed by a Chi-Square test. As before, the level of significance used was.05, and a significant dependency between the groups was reported when p < 0.05 while no significant dependency was reported when p > 0.05.

## Results

Among the 102 patients with primary inguinal hernia, 52 underwent MPR, 50 underwent LR, and all received a prolene graft. The same surgeon operated on all patients at the same hospital.

When demographics and patient characteristics were analyzed, the mean age was 26.3 years in the MPR group, and all patients were males. Mean BMI was 26.4 kg/m^2 ^with a general ASA score of one, and the patients had mostly Nyhus type two indirect inguinal hernias on the right side. Among the individuals who underwent LR, the mean age was 30.4 years and there were 49 males and one female patient. Mean BMI was 28.8 kg/m^2^ with a general ASA score of one, and the patients had mostly Nyhus type two indirect inguinal hernias on the right side. In the MPR group, three patients received general anesthesia, and 49 underwent spinal anesthesia, while three received general, and 47 received spinal anesthesia in the LR group.

No statistically significant differences were found between the two groups when two different techniques were used regarding gender, age, BMI, ASA score, comorbidity, Nyhus type of hernia, and hernia site (p > 0.05). No significant associations were found between the technique used and complications, such as wound infection and scrotal hematoma/seroma (p > 0.05)

When the duration of operation was evaluated, the mean duration of operation was found to be significantly higher in the LR group compared with that in the MPR group (p < 0.05). When the duration of hospitalization was evaluated, mean duration of hospitalization was found to be significantly higher in the LR group compared to the MPR group (p > 0.05) (Table 1).

**Table 1:** Comparison of operative time and hospitalizasyon time values

		Technique Used					Mann Whitney U Test
		n	Mean	Min.	Max.	SD	p
Operation time	Mesh plug	52	31.92	25	60	6.27	<.05
	Lichtenstein	50	45.50	30	65	8.47	
	Total	102	38.58	25	65	10.06	
Hospitalization time	Mesh plug	52	1.46	1	4	.64	<.05
	Lichtenstein	50	3.10	1	8	1.58	
	Total	102	2.26	1	8	1.45	

Evaluation of postoperative first-day pain values revealed that mean VAS was significantly higher in the LR group compared with that of the MPR group (p < 0.05).  Evaluation of postoperative first-week pain values revealed that the mean VAS was significantly higher in the LR group compared to the MPR group (p < 0.05). Evaluation of postoperative sixth month pain values revealed that the mean VAS was significantly higher in the LR group compared with that of the MPR group (p < 0.05), (Table 2).

**Table 2:** Comparison of postoperative pain values

Time		Pain values (VAS score)						Mann Whitney U Test
		Technique Used	n	Mean	Min.	Max.	SD	p
Post Operative 1st Day Pain	Mesh Plug		52	2.33	1	5	1.12	<.05
	Lichtenstein		50	3.86	2	6	.88	
	Total		102	3.08	1	6	1.26	
Post Operative 1st Week Pain	Mesh Plug		52	1.02	0	4	.78	<.05
	Lichtenstein		50	2.08	0	3	.85	
	Total		102	1.54	0	4	.97	
Post Operative 6th Month Pain	Mesh Plug		52	.33	0	2	.58	.028
	Lichtenstein		50	.60	0	2	.70	
	Total		102	.46	0	2	.66	

Post-operative pain status was evaluated by asking the patient and scoring VAS score. When decrease in pain was evaluated, the mean decrease in pain in the MRP group was significantly higher compared with the mean decrease in pain in the LR group (p < 0.05) (Table 3).

**Table 3:** Comparison of the decrease in pain status

	Pain reduction process (VAS score)						Mann Whitney U Test
	Technique used	n	Mean	Min.	Max	SD	p
Decreased amount of pain	Mesh plug	52	2.00	1	5	1.05	<.05
	Lichtenstein	50	3.26	1	5	.90	
	Total	102	2.62	1	5	1.16	

The averages of returning to normal activity in the MPR and the LR groups were 21 and 25 days. When determining the return to normal activity, the patients were asked when they could do daily work after surgery. Although patients in the MPR group returned to normal activity quicker than patients in the LR group, no statistically significant difference was found between the groups (p > 0.05). No recurrence was found after the follow-up period of two years in the MPR group. Recurrence was found in only one patient in the LR group. No statistically significant difference was found in recurrence between the two technical repair groups (p > 0.05)

## Discussion

Currently, inguinal hernia operations have been performed as laparoscopic posterior mesh repair and open anterior mesh repair techniques. The aim is to perform the operation with the lowest risk of recurrence and to decrease the postoperative discomfort of the patient to a minimal level [1].

In general, the most frequently used technique in the treatment of primary inguinal hernia is open anterior mesh repair. In 1986, Lichtenstein was the first to report tension-free mesh repair in adults for inguinal hernia repair [2]. LR became a standard procedure since it had a lower rate of recurrence compared to other conventional suture repair techniques. Subsequently, different tension-free methods such as mesh plug and Kugel mesh techniques were developed in 1993 [3]. Mesh is placed more easily in MPR, and the duration of the operation is thus shorter. Mesh plugs in different figures and designs have been produced. The MPR technique has been performed widely, especially in Japan [4].

In this randomized prospective study, various aspects of both the LR and the MPR methods in the open anterior surgical treatment of inguinal hernia were compared.

Various investigators, such as Dalenbäck and Zhao [5], reported no difference in complications, chronic pain, return to work, and recurrence between MPR and LR; however, they reported a remarkably shorter duration of operation for MPR compared with that for LR [4]. They stated that the surgeons both learned and performed MPR more easily [4, 6]. When the duration of operation was evaluated in this study, MPR seems to be shorter and more advantageous compared with LR regarding the duration of operation. However, when the duration of operation was 31.9 minutes was evaluated in the literature, it was found to vary between 20 and 50 minutes [6].

The duration of hospitalization was reported to be similar in various studies comparing MPR and LR [7-9]. When the duration of hospitalization was analyzed in this study, the MPR technique resulted in a shorter hospitalization, which is more advantageous. In this study, the duration of hospitalization in the MPR group was 1.46 days and is compatible with the literature.

The most important postoperative complication following open anterior mesh repair is chronic pain in general [6]. Chronic pain is seen in approximately 1% - 31% of cases and affects patients’ quality of life [6-7]. Mechanical triggers, such as trauma to the ilioinguinal and genitofemoral nerves and cicatrization, are the main causes of chronic pain following inguinal hernioplasty [7-8]. In addition, psychological issues may have an adverse effect on the condition [8-9]. In some studies, less chronic pain was reported in MPR than in LR; and in some studies the opposite is said [10-12]. In this study, the inguinal pain level on day one, week one, and month six was significantly lower in the MPR group compared with that in the LR group. When the groups with different techniques were compared, the decrease in pain occurred faster in the MPR group compared with that in the LR group.

In some studies, lower rates of both complications and pain have been reported to develop following MPR [11,13]. In contrast, the complication rate was reported to be higher especially due to migration of the mesh [4,14,15]. Intestinal volvulus, obstruction, and perforation are reported complications due to mesh migration in MPR different from LR [9,16]. However, no statistically significant difference was reported regarding complications between MPR and LR in most studies performed [5-9].

Antibiotic prophylaxis has been demonstrated to be effective in the prevention of surgical field infections [17]. We did not routinely use antibiotics in this study and a wound infection problem developed in two patients.

No significant differences have been reported between MPR and LR regarding recurrence. In the present study, no significant difference was reported between MPR and LR in terms of recurrence similar to the data in the literature [6,7,18].

Laparoscopic posterior mesh repair techniques have been reported currently to be superior to both MPR and LR procedures concerning postoperative pain, duration of hospitalization, and duration of healing [19]. However, MP repair is expected to retain its place in the treatment of hernias due in part to its low cost, short learning period, no requirement for special equipment, and its ability to be performed using local anesthesia if preferred by the patient [19-21]. It was noted that the low cost was determined, including the need for surgical equipment and supplies, time of departure from hospital, duration of daily return to life, complication and relapse status. Mesh plug insertion is faster, cheaper, technically easier, does not require general anesthesia, and is suitable to be done by surgeons as part of their general practice without special instruments and by junior surgeons [19]. Some studies say the opposite. These studies results indicate that laparoscopic hernioplasty is superior to tension-free open herniorrhaphy with mesh-plug and patch or Lichtenstein’s operation in terms of postoperative pain and rehabilitation [20].

In the literature, other factors associated with chronic pain, such as gender, activity level, and mental condition, were reported to have no effect on the prevalence of chronic pain. Chronic pain has a multifactorial etiology and may develop despite meticulous handling of nerves and impeccable surgical technique [22]. Recently performed studies reported low rates of chronic pain, early complications, and recurrence rates for both laparoscopic repair and MP repair six, 12, and 24 months after surgery with no statistically significant difference between the two techniques [23].

Currently, the main principle in the treatment of inguinal hernia is to prefer methods that are easy to apply; have low morbidity, shorter duration of hospitalization, lower cost, less postoperative pain, and shorter return to normal daily activities; and work schedules with minimal rates of both complication and recurrence [1, 6, 22]. In this study, we compared MPR and LR in patients who were diagnosed with indirect inguinal hernia. Although MPR is not superior to LR regarding recurrence and complications, we observed that MPR had a significant superiority compatible with the literature. This superiority is regarding postoperative pain, quicker recovery, shorter duration of hospitalization, broader applicability to patients of any age, shorter duration of operation, and ways it affects the quality of life of the patient and procedural comfort of the surgeon [12, 24]. Therefore, we suggest that the MPR technique may be the preferred option for patients with indirect inguinal hernia.

## Conclusions

Although several surgical techniques for the repair of indirect inguinal hernia have been defined, there is still no ideal method of treatment due to recurrence rates and other postoperative complications. Thus, currently used surgical procedures remain fodder for debate. In the present study, MPR repair was observed to be safe, simple, and easily applicable without any differences in development of complications, chronic pain, and recurrence. We suggest that MPR is easily applied, shortens the duration of both operation and hospitalization, and is superior to other techniques regarding postoperative pain, quality of life, and low cost as a result of all these. However, it is best to pay attention to patient selection and select the technique accordingly.
